# *julius seizure* acts within *teashirt* -expressing neurons to protect *Drosophila melanogaster* from bang-sensitivity

**DOI:** 10.17912/micropub.biology.002103

**Published:** 2026-06-04

**Authors:** Derek M. Dean, Lillian E. Codd, Mira Eras, Katherine Lopez, Huy Pham, Dylan Taylor, Aisha Abakura, Zoe Gabriel, Alyana J. Granados, Ben Litvak-Hinenzon, Jeremy Wen, Catalin Yturralde, David L Deitcher

**Affiliations:** 1 Biology, Williams College, Williamstown, Massachusetts, United States; 2 Neurobiology and Behavior, Cornell University, Ithaca, New York, United States

## Abstract

Loss of function in the *Drosophila melanogaster* gene *julius seizure* ( *jus* ) causes the fly to have a seizure-like condition known as bang-sensitivity. Here, we characterize the expression patterns of two different *jus* reporters, a *jus* -GAL4 construct and a *jus* protein trap allele, in the central nervous system during the developmental stages that *jus* expression is required. The majority of cells that expressed the *jus* -GAL4 construct co-expressed a GAL80 enhancer trap in the homeotic gene *teashirt* ( *tsh* ); expression of *jus* in this subpopulation was necessary to prevent bang-sensitivity.

**Figure 1. (A-D) Confocal microscope imaging of the expression of two different julius seizure ( jus ) reporters in the central nervous system (CNS) during the critical period for jus expression, (E-H) higher magnification examples of jus reporter colocalization, (I-K) comparison of jus -GAL4 expression to that of teashirt ( tsh )-GAL80 and tsh -GAL4 constructs, and (L) bang sensitivity of jus -GAL4 > jus RNAi, jus -GAL4 > jus RNAi + tsh -GAL80, and tsh -GAL4 > jus RNAi flies:** Top left, schematic of a neuronal cell body, indicating the colors emitted by the two *jus* expression reporters that were used in this study. Magenta nuclei are from *jus* -GAL4 driving UAS-nls-mCherry expression (“ *jus* -GAL4 > nls-mCh”), green cell bodies express the Jus ^GFSTF^ fusion protein, and colocalization between these two expression patterns either appears as a magenta dot within a green cell, or as yellow if the colors overlap. A legend lists the symbols and abbreviations used in **(A-K)** . White scale bar in each confocal image indicates 100 µm. During pupal stages P2 **(A)** and P4 **(B)** , *jus* -GAL4 > nls-mCh and Jus ^GFSTF^ were both expressed widely in the ventral nerve cord (VNC), with some expression across the central brain (CB), and virtually no expression in the optic lobes (OL). Some colocalization was seen in the VNC, particularly in cells along the midline and lateral margins of the posterior segments. The head cuticle (c) tended to autofluoresce green. **(C)** By stage P5ii, and through to the end of the critical period **(D)** , *jus* -GAL4 > nls-mCh expression was strongest in the most posterior and anterior VNC segments, while Jus ^GFSTF^ expression was most prominent in the mid-thoracic region of the VNC; colocalization of the two signals was seen in a narrow, lateral band of cells that wrapped around the mid-thoracic ganglia. Fat body (f) often autofluoresced green. At the transition between P6 and P7 **(D)** , additional colocalization was seen in a band of cells across the protocerebrum (PR), a region of the brain that receives and processes information from the optic lobe. **(E)** and **(F)** show higher magnification examples of nls-mCh/GFP colocalization in the VNC. **(F)** is a transverse section of an abdominal segment to show colocalization on the dorsal (top) as well ventral (bottom) margins of the VNC. **(G)** shows a higher magnification example of colocalization in a lateral band across the mid-thorax. **(H)** is a higher magnification view of colocalization in the protocerebrum (PR) as well as a small number of cells in the central brain (CB). **(I)** *jus* -GAL4 > nls-mCh expression in the CNS of a P4 pupa that did not carry *jus ^GFSTF^* . As in panel **B** , mCherry signal was seen throughout the VNC. Even in the absence of Jus ^GFSTF^ , the head cuticle (c) and fat body (f) strongly fluoresced green, confirming that these were non-specific, autofluorescent signals. **(J)** The number of *jus* -GAL4 > nls-mCh-expressing nuclei was significantly reduced in pupae that also carried the *tsh* -GAL80 construct. Loss of nls-mCh signal was particularly noticeable throughout the VNC. This particular specimen also happens to have reduced green autofluorescence, but this was not specific to the *jus* -GAL4 > nls-mCh + *tsh* -GAL80 genotype, *i.e.* , cuticle and fat body autofluorescence varied widely in all sample groups [compare panels **(B)** , **(I)** , and **(J)** ]. **(K)** *tsh* -GAL4 > nls-mCh expression was ubiquitous in the VNC, posterior central brain, and compound eyes (CE). **(L)** When *jus* -GAL4 was used to express UAS- *jus* RNAi (“ *jus* -GAL4 > *jus* RNAi”), 98.0% of flies were bang-sensitive. *tsh* -GAL80 suppressed *jus* -GAL4 > *jus* RNAi bang-sensitivity down to 36.6%. UAS- *jus* RNAi expression using a *tsh* -GAL4 driver (“ *tsh-GAL4* > *jus* RNAi”) caused 100% bang-sensitivity. n = 150 or more for each sample group. Two-tailed Fisher’s exact tests: p < 1.1e-110 ( *jus* -GAL4 > *jus* RNAi vs. RNAi-only and GAL4-only controls) p = 1.1e-43 ( *jus* -GAL4 > *jus* RNAi vs. *jus* -GAL4 > *jus* RNAi + *tsh* -GAL80), and p < 2.1e-89 ( *tsh* -GAL4 > *jus* RNAi vs. RNAi-only and GAL4-only controls).

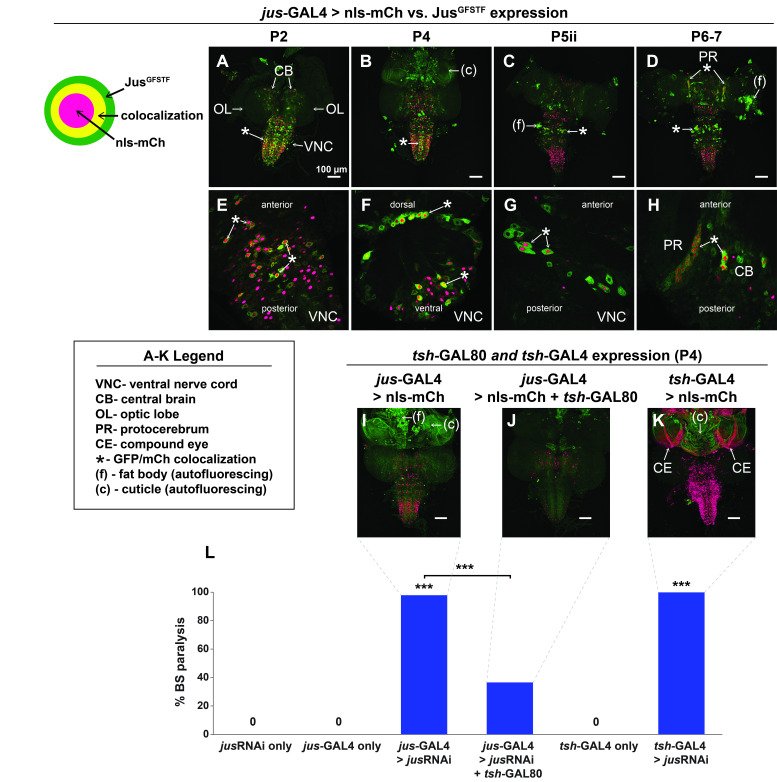



## Description

The fruit fly *Drosophila melanogaster* has been an informative model system for studying seizure disorders. Mutations in certain genes cause *Drosophila* adults to have seizure-like neural activity and muscle spasms, followed by a period of paralysis before recovery. This behavioral sequence can be induced by external stimuli, including extreme temperature changes; electrical shock; strobe lighting; and mechanical shock, for example from shaking the culture vial upon a lab vortex machine. Given the ease and effectiveness of mechanical shock, fly mutants with this seizure-like phenotype are considered “bang-sensitive” (Baraban, 2007; Parker et al., 2011a; Dean et al., 2018; Mituzaite et al., 2021; Fischer et al., 2023).

Most loci that affect bang-sensitivity are associated with basic neurophysiological function. For examples, *paralytic* ( *para* ) encodes a subunit of a voltage gated Na ^+^ channel, and *ATPα* encodes a subunit of Na ^+^ /K ^+^ -ATPase (Schubiger et al., 1994; Parker et al., 2011b). In contrast, *julius seizure* ( *jus* , formerly known as *slamdance* and *sda* ) has a more cryptic role in regulating neural activity because, other than two predicted transmembrane spans that bracket a cysteine-rich, extracellular loop, the Jus protein has no conserved domains that imply a specific function (Horne et al., 2017; Dean et al., 2018). In preprint, we have reported that Jus physically associates with 23 other proteins, including ATPα and Nrv3, which are α and β subunits of the Na+/K+-ATPase, so it is possible that Jus affects the assembly, positioning, stability, and/or activity of a channel that is essential for maintaining resting membrane potential (Bermudez et al., 2023). In two separate experiments, we had used RNAi to knock down *jus* transcript at specific developmental stages and found evidence that *jus* expression may be required as early as the wandering larval phase and as late as pupal stage P7, with a peak requirement at about P4-5, when the adult head epidermis everts and surrounds the brain (Horne et al., 2017; experiment replication/refinement described in preprint Bermudez et al., 2023). We also found that *jus* expression is required in neurons—most significantly in cholinergic neurons—and not in glia, to protect against bang-sensitivity (Horne et al., 2017; Dean et al., 2018). With this as a starting point, our goals were (1) to determine the expression pattern of *jus* during its critical period for expression, and (2) to begin to characterize the spatial distribution of neurons that must express *jus* to protect the adult against bang-sensitivity.

We used two reporters to assess *jus* expression in the pupal CNS: (1) *jus ^GFSTF^* is a protein trap allele that inserts an EGFP-FlAsH-StrepII-TEV-3xFlag multi-tag cassette in the putative extracellular domain of Jus; this fusion protein mislocalizes to the cytoplasm of neuronal cell bodies, and its expression can be detected by GFP fluorescence (Diao et al., 2015; Dean et al., 2018). (2) GMR90B09-GAL4, hereafter referred to as *jus* -GAL4, is a construct that uses the *jus* promoter region to drive GAL4 expression (Jennet et al., 2012; Bermudez et al., 2023). A GAL4 construct that reflects endogenous *jus* expression would be expected to cause severe bang-sensitivity when used to express UAS- *jus* RNAi. Retesting *jus* -GAL4 for this study, we found that, while GAL4-only and RNAi-only controls were not bang-sensitive, 98% of *jus* -GAL4 > *jus* RNAi flies were, confirming that this *jus* -GAL4 construct is expressed in neurons that express *jus* and that affect bang-sensitivity ( Figure 1L ).

To compare the expression patterns of *jus* -GAL4 and Jus protein, *jus* -GAL4 > nls-mCh and *jus ^GFSTF^* stocks were mated, and progeny were examined during metamorphosis. Throughout the critical period for *jus* expression, both nls-mCh and GFP were expressed widely in the ventral nerve cord (VNC) and somewhat in the central brain, with little expression in the optic lobes ( Figure 1A- D). Despite the similar spatial distribution of nls-mCh and GFP signals across the nervous system, few cells strongly expressed both nls-mCh and GFP, with most examples located in the VNC, and later in the protocerebrum, a region of the central brain that directly associates with the optic lobe ( Figure 1E- H). There are several possible explanations for the apparent mismatch between the *jus* -GAL4 > nls-mCh and Jus ^GFSTF^ expression patterns: 1) *jus* -GAL4 > nls-mCh may not fully reflect endogenous *jus* expression, because although the *jus* -GAL4 construct includes the entire *jus* promoter region, it may be missing enhancer sequences that refine the expression pattern. 2) Jus ^GFSTF^ may not fully reflect wild type Jus ^+^ protein expression, because the mutant protein mislocalizes from the axon to the cell body (Dean et al., 2018), suggesting it is structurally defective. Therefore, Jus ^GFSTF^ stability/turnover rate may differ from that of Jus ^+^ protein. 3) *jus* -GAL4 > nls-mCh and Jus ^GFSTF^ expression may overlap more significantly than suggested by confocal microscopy, but much of this overlap may have been undetected due to differing expression levels or protein turnover kinetics of nls-mCh vs. Jus ^GFSTF^ in any given cell. Regardless of which of these hypotheses is correct, *jus* -GAL4 > *jus* RNAi caused very high rates of bang-sensitive paralysis, so *jus* -GAL4 must be expressed in neuronal subpopulations that strongly affect bang-sensitivity.

Given the broad expression of *jus* -GAL4 and Jus ^GFSTF^ across the VNC, we considered whether a GAL80 construct associated with *teashirt* ( *tsh* ), a homeotic gene that directs development of the VNC, might be expressed in *jus* -GAL4 > nls-mCh-labeled cells (Röder et al., 1992; Rubio-Ferrera et al., 2023). If *tsh* -GAL80 and *jus* -GAL4 > nls-mCh expression patterns overlap, *tsh* -GAL80 would be expected to block GAL4 function, reducing the number of mCh-labelled nuclei. Consistent with this prediction, *jus* -GAL4 > nls-mCh + *tsh* -GAL80 pupae, throughout the *jus* critical period, had a low number of mCh-labelled nuclei in the VNC ( Figure 1I vs. J). Quantifying at P4, the peak of the critical period, it was found that *jus* -GAL4 > nls-mCh + *tsh* -GAL80 central nervous systems had 29.9% the average number of labeled nuclei seen in *jus* -GAL4 > nls-mCh controls (averages of 113.4 ± 7.7 SEM vs. 379.6 ± 26.5 SEM, n=9 for each group; p = 3.6e-6, Student’s t-test). *tsh* -GAL4, a construct swapped into the same enhancer trap sequence as *tsh* -GAL80, was used to express UAS-nls-mCh. Throughout the critical period, *tsh* -GAL4 > nls-mCh was ubiquitously expressed across the VNC, with some labeling within the posterior section of the central brain, and high expression in the compound eyes ( Figure 1K ).

Finally, we tested if *jus* expression is required in *tsh* -GAL80- and *tsh* -GAL4-expressing cells to prevent bang-sensitivity. In a previous study, *tsh* -GAL80-expressing neurons were shown to protect against seizure-like behavior in *Drosophila* larvae, using an optogenetic model and electrical shock (Giachello and Baines, 2015). In our experiments, *tsh* -GAL80 strongly suppressed the bang-sensitivity of *jus* -GAL4 > *jus* RNAi flies (36.6% with *tsh* -GAL80 vs. 98.0% without; Figure 1L ), and when *tsh* -GAL4 was used to express *jus* RNAi, 100% of flies were bang-sensitive ( Figure 1L ; the end of the figure caption discusses statistics).

Considering the involvement of *tsh* -GAL80- and *tsh* -GAL4-expressing neurons in regulating *jus* -associated bang-sensitivity, along with the persistent expression of these markers in the pupal VNC, an intriguing possibility is that bang-sensitivity can be caused by loss of *jus* expression in neural tissue outside of the brain. However, given that *tsh* -GAL4 seems to be expressed in the central brain, at least to some extent ( Figure 1K ), it is certainly possible that *jus* expression in the central brain is what protects the fly against bang-sensitivity, and that *jus* expression in the VNC has a supporting or distinct function. Future experiments could test these two hypotheses, and otherwise spatially and functionally characterize the *jus* -expressing neurons that affect bang-sensitivity.

## Methods

**Fly stocks, diet, and experimental crosses:** The fly strains that were used in our experiments are listed below. Stocks received from the B loomington *D rosophila* S tock C enter are denoted by “BDSC [stock number]”.

● ***jus* -GAL4:** *w ^1118^* ; P{ *y ^+t7.7^* *w ^+mC^* =GMR90B09-GAL4}attP2 (BDSC 607421; Bermudez et al., 2023)

● **Control strain for *jus* -GAL4 background:** *w ^1118^* (BDSC 6326)

● **UAS- *jus* RNAi:** *y ^1^* *v ^1^* ; P{ *y ^+t7.7^* *v ^+t1.8^* =TRiP.JF03192}attP2 (BDSC 28764; Perkins et al., 2015)

● **Control strain for *jus* RNAi background:** *y ^1^* *v ^1^* ; P{ *y ^+t7.7^* =CaryP}attP2 (BDSC 36303; Groth et al., 2004)

● ***jus ^GFSTF^* :** *w* ; *Kr* /CyO; *jus ^GFSTF^* /TM6C *Sb Tb* (BDSC 607422; Dean et al., 2018). For our experiments, the *Kr* and CyO second chromosomes had been crossed out of the background, leaving a strain with the genotype *w* ; *+* /+; *jus ^GFSTF^* /TM6C *Sb Tb*

● **UAS-nls-mCh (for crosses involving *jus* -GAL4)** : w; P{ *w ^+mC^* =UAS-mCherry.NLS}3 (BDSC 38424; Caussinus et al., 2008)

●A ***jus* -GAL4 > nls-mCh** stock was made by recombining the *jus* -GAL4 and UAS-nls-mCh constructs described above onto the same chromosome and a *w ^1118^* ; *jus* -GAL4 UAS-nls-mCh strain was made.

● ***jus* -GAL4** ***jus ^GFSTF^* :** This stock was generated by recombining *jus* -GAL4 and *jus ^GFSTF^* onto the same chromosome and balancing over TM6C. Full genotype was *w ^1118^* ; *jus* -GAL4 *jus ^GFSTF^* /TM6C *Sb Tb.*

● ***tsh* -GAL80:** w; P{ y ^+t*^ =GAL80}tsh[md621-GAL80]/CyO; TM2/TM6B (BDSC 605556; Clyne and Miesenbock, 2008). For our experiments, the *tsh* -GAL80 construct was rebalanced over the CyO, P{Wee-P.ph0}Bacc[Wee-P20] chromosome from BDSC 52267; this CyO balancer ubiquitously expresses GFP, enabling us to identify pupae that carried the *tsh* -GAL80 chromosome (Clyne et al., 2003).

● ***tsh-* GAL80; *jus* -GAL4:** A strain carrying both the *jus* -GAL4 and *tsh* -GAL80 constructs was made. Full genotype: *w ^1118^* ; P{ y ^+t*^ =GAL80}tsh[md621-GAL80]/CyO; P{ *y ^+t7.7^ w ^+mC^* =GMR90B09-GAL4}attP2

● ***tsh* -GAL4:** *y ^1^* *w ^1118^* ; P{ *w ^+mW.hs^* =GawB}tsh[md621]/CyO; P{ *w ^+mC^* =UAS-y.C}MC1/TM2 (BDSC 3040; Soller et al., 2006)

● **UAS-nls-mCh (for crosses involving *tsh* -GAL4)** : w; P{ *w ^+mC^* =UAS-mCherry.NLS}2 (BDSC 38425; Caussinus et al., 2008). This strain was crossed to *tsh* -GAL4 to generate the image in Figure 1K, while crosses involving *jus* -GAL4 and mCherry used the related UAS-nls-mCh stock described above (BDSC 38424).

Larvae and flies were fed a modified yeast/dextrose/cornmeal diet (Dean et al., 2016; Dean et al., 2020). Parental stocks were maintained at room temperature, while experimental crosses were incubated at 25°C as the progeny developed. Mating schemes for experimental crosses were as follows (see stock list immediately above for full parental genotypes):

● **Figure 1A- H (Comparing *jus* -GAL4 > nls-mCh and *jus ^GFSTF^* expression in CNS)**

*jus* -GAL4 > nls-mCh females X *jus ^GFSTF^* males

● **Figure 1I- K (Expression of *tsh* -GAL80 and *tsh* -GAL4 constructs in CNS)**

Figure 1I : *jus* -GAL4 > nls-mCh females X *w ^1118^* males

Figure 1J : *jus* -GAL4 > nls-mCh females X *tsh* -GAL80/CyO, P{Wee-P.ph0}Bacc[Wee-P20] males. Progeny carrying *tsh* -GAL80 were identified by a lack of GFP expression from the CyO, P{Wee-P.ph0}Bacc[Wee-P20] balancer (Clyne et al., 2003).

Figure 1K : *tsh* -GAL4 females X nls-mCh (BDSC 38425) males

● **Figure 1L (Bang-sensitive assays)**

*jus* -RNAi-only control: *w ^1118^* females X UAS- *jus* RNAi males

*jus* -GAL4-only control: *jus* -GAL4 females X *y ^1^* *v ^1^* ; P{ *y ^+t7.7^* =CaryP}attP2 (BDSC 36303) males

*jus* -GAL4 > *jus* RNAi: *jus* -GAL4 females X UAS- *jus* RNAi males

*jus* -GAL4 > *jus* RNAi with *tsh* -GAL80: *tsh-* GAL80; *jus* -GAL4 females X UAS- *jus* RNAi males

*tsh* -GAL4-only control: *tsh* -GAL4 females X *y ^1^* *v ^1^* ; P{ *y ^+t7.7^* =CaryP}attP2 (BDSC 36303) males

*tsh* -GAL4 > *jus* RNAi: *tsh* -GAL4 females X UAS- *jus* RNAi males

**CNS preparation for confocal microscopy:** Wandering larvae and pupal stages P1-P7 were identified by morphological criteria (Bainbridge and Bownes, 1981; Chyb and Gompel, 2013). Each CNS was dissected in cold PBS, fixed in PBS + 4% formaldehyde for 30 minutes at room temperature with gentle rocking, then rinsed twice in PBS + 0.3% Triton X-100 (all chemicals obtained from Sigma-Aldrich).

CNS tissue was transferred to microscope slides and overlaid with Vectashield Antifade Mounting Media (Vector Labs), then with a #1.5 18 x 18 mm coverslip (Fisher Scientific). Coverslip edges were sealed with nail polish. Slides were stored in the dark at 4ºC until they were imaged at the Williams College confocal microscope facility.

**Confocal microscopy and post-production image processing:** Within a week after slide preparation, pupal CNS tissue was imaged using a Nikon Ti2 inverted microscope and Nikon A1R HD software.

Z stack series were collected with the following settings: 20X objective lens, 2 µm steps, 32X averaging resonant scan, 0.9 pinhole, 1024 x 1024 pixel resolution, and power/off-set/gain settings were FITC 25/0/43 and TRITC 0.8/0/65. To reduce background from cuticle and fat body at the later pupal stages, FITC power was decreased to as low as 4.5 while holding other settings constant. Z-series were saved as ND2 files. Each Figure 1 image was made by merging a Z-series into a focused EDF document and saving as an uncompressed TIFF.

For quantification of *jus* -GAL4 > nls-mCh-labeled nuclei at the P4 stage (genotypes shown in Figure 1I- J), Z stack series were captured with similar settings to those described above, but a 10X objective lens was used, and power/off-set/gain settings were FITC 4.5/0/43 and TRITC 0.4/0/61. Sections were collectively exported into a multipage TIFF file, imported into Fiji, color channels were split, the TRITC channel was selected, and 3D objects were counted using a threshold of 800 and a minimum size of 35, parameters which appeared to reliably identify each visible nucleus.

**Bang-sensitivity assays:** 3-7-day-old adult flies were vortex assayed for bang-sensitive paralysis based on a previously established protocol (Bermudez et al., 2023). Adult progeny of experimental crosses were allowed to emerge over 4 days, anesthetized with CO _2_ , transferred to glass vials containing fresh diet, and vials were capped with plastic foam plugs (Fisher Scientific)—we specify the container materials because, in our experience, bang-sensitivity was enhanced if flies were vortexed in glass vials with foam plugs compared to standard plastic vials and cotton plugs, perhaps because the latter materials would have softer impact on the flies during vortexing. Vials were stored at room temperature for 3 additional days; during this period, to avoid making the flies refractory to bang-sensitive paralysis, vials were not jostled or otherwise moved (Pavlidis and Tanouye, 1995). After recovery, each vial was gently picked up then pressed upside down against a Vortex Genie (Sigma-Aldrich) on maximum setting for 10 seconds to agitate the flies against the cap and vial wall. Immediately afterwards, the number of paralyzed flies were counted and compared to the total number of living flies in the vial.

**Image editing, data analysis, and figure generation:** The focused EDF TIFF files from confocal imaging were imported into Adobe Photoshop 2021. Exposure was increased +1, and to increase accessibility for red/green colorblind readers, the red mCh channel was converted to magenta by adding a Hue/Saturation Adjustment Layer and altering the hue of the reds -35 (Summerbell, 2019). Otherwise, no image adjustments were made. Vortex data were graphed and analyzed using JMP Student Version 18. The Figure 1L graph was saved as an editable JPG. Photos and the graph were assembled into a figure and annotated using Adobe Illustrator 2021.

**AI statement:** No AI was used in this project for data collection, data analysis, writing, figure generation, or editing.
